# Anticancer Activity of Green Synthesized ZnO Nanoparticles from *Ficus benghalensis* Bark in Osteosarcoma Cells

**DOI:** 10.3390/nano16161006

**Published:** 2026-08-16

**Authors:** Essa M. Sabi, Khalid M. Sumaily, Musaad B. Alsahly, Noura H. Mojammamy, Ahmed H. Mujamammi, Nouf O. AlAfaleq

**Affiliations:** 1 Clinical Biochemistry Unit, Department of Pathology, College of Medicine, King Saud University, Riyadh 11461, Saudi Arabia; ksumaily@ksu.edu.sa (K.M.S.); amujamammi@ksu.edu.sa (A.H.M.); 2 Department of Physiology, College of Medicine, King Saud University, Riyadh 11149, Saudi Arabia; malsahly@ksu.edu.sa; 3 Clinical Trial Section, Department of Executive Administration of Academic Affairs, King Saud University Medical City, King Saud University, Riyadh 11149, Saudi Arabia; nmojammamy@ksu.edu.sa; 4 Department of Biochemistry, College of Science, King Saud University, Riyadh 11451, Saudi Arabia; nalafaleg@ksu.edu.sa

**Keywords:** *Ficus benghalensis*, zinc oxide nanoparticles, osteosarcoma, apoptosis, reactive oxygen species, oxidative stress, anticancer

## Abstract

Osteosarcoma is the third most common malignancy among children and adolescents, necessitating the development of effective therapeutic strategies. This study investigated the anticancer activity of green-synthesized zinc oxide nanoparticles (ZnO-NPs) fabricated using bark extract of *Ficus benghalensis* against the human osteosarcoma Saos-2 cell line. The synthesized ZnO-NPs were characterized using UV–Vis spectroscopy, Fourier Transform Infrared (FTIR), and X-ray Diffraction (XRD), confirming nanoparticle formation and a hexagonal wurtzite crystalline structure. Cytotoxicity evaluation revealed significant dosage-dependent inhibition of Saos-2 cell proliferation, with an IC_50_ value of 75 μg mL^−1^. Morphological alterations and 4′,6-diamidino-2-phenylindole (DAPI) staining confirmed apoptotic cell death following ZnO-NPs treatment. Furthermore, ZnO-NPs induced oxidative stress by increasing nitric oxide (NO) and lipid peroxidation (LPO) levels while significantly reducing antioxidant markers, including catalase (CAT), superoxide dismutase (SOD), and glutathione (GSH). Flow cytometry analysis demonstrated G0/G1cell cycle arrest, accompanied by elevated caspase-8 activity. Gene expression analysis showed upregulation of Bax and p53 and downregulation of Bcl-2, indicating activation of the mitochondrial apoptotic pathway. Collectively, these findings demonstrate that phytochemical-mediated ZnO-NPs exert potent anticancer effects against Saos-2 cells through oxidative stress-induced apoptosis and cell-cycle arrest, highlighting their potential for osteosarcoma therapy.

## 1. Introduction

Osteosarcoma is typically a high-grade tumor with a great risk of developing lung metastases [1,2]. The disease predominantly affects children, adolescents, and young adults during the phase of skeletal development. The widely recognized conventional treatment options for osteosarcoma are the surgical approach, chemotherapy, and radiotherapy. Despite improvements in radiation and chemotherapy, the core problem with these therapies is that they possess adverse outcomes for the patients by damaging healthy cells while destroying cancer cells, resulting in a 5-year survival rate of approximately 60–80% for patients with localized disease. Additionally, they are limited by minimal solubility, lack of specific targeting, and development of resistance, inflicting considerable undesirable effects on the immune system and thereby reducing survival probability. Nonetheless, the prognosis is constrained for patients with recurrent, metastatic, or chemo-resistant osteosarcoma, suggesting the urgent need for novel therapeutic strategies [3].

A newer path of interdisciplinary research spanning biology, engineering, and medicine has emerged through cancer nanotechnology, primarily focusing on advancing cancer diagnosis and therapeutic techniques. Nanoparticles possess unique physicochemical attributes, including high surface-area-to-volume ratios, tunable size and morphology, improved cellular uptake, and the potential to selectively accumulate within tumor cells. These traits facilitated their applications in numerous fields, especially in targeted drug delivery and cancer therapy [4,5,6]. Among nanoparticles, metal oxide nanoparticles garnered prominence by virtue of their excellent cytotoxicity. ZnO-NPs are one such metal oxide nanoparticle with tremendous antimicrobial, anti-diabetic, anti-inflammatory, and anticancer potential. ZnO-NPs tend to be less hazardous and more economically viable than several other metal oxide nanoparticles. Owing to their enhanced biocompatibility, they are recognized by the U.S. Food and Drug Administration as being Generally Recognized as Safe (GRAS), supporting their application as therapeutic agents and in drug formulations. Likewise, ZnO-NPs can also be developed as diagnostic agents, as they can be utilized in bioimaging applications [7,8,9,10,11].

The fabrication method substantially impacts the biological activity and safety of ZnO-NPs. The downsides of chemical and physical fabrication methods include the need for sophisticated equipment, high-pressure or temperature requirements, the generation of harmful wastes, and energy consumption during the process. Conversely, biological methods offer inexpensive and environmentally sustainable means of synthesizing nanoparticles [12,13,14,15,16]. Plant-mediated synthesis has gained popularity due to the presence of phytocompounds, including polyphenols, flavonoids, tannins, alkaloids, and terpenoids, which act as natural reducing, stabilizing, and capping agents during nanoparticle formation. This phyto-mediated approach improves the biocompatibility of the synthesized nanoparticles. The genus *Ficus* is generally distributed in tropical and subtropical areas all over the world. *Ficus benghalensis* is well-known for its exceptional therapeutic qualities, possessing antibacterial, anti-inflammatory, anti-diabetic and antitumor activities. The parts of the tree were traditionally used in folk medicine to treat skin and urinary infections, dysentery, and diarrhea [12,17,18]. However, there are plenty of studies reporting the anticancer activity of ZnO-NPs and the use of plant-mediated synthesis strategies; limited data are available on the cytotoxic potential of *F. benghalensis*-mediated ZnO-NPs against osteosarcoma cells. Hence, in the current study, ZnO-NPs are fabricated by a phytogenic method using bark extract of *Ficus benghalensis* and further evaluated for cytotoxicity on the Saos-2 osteosarcoma cell line.

## 2. Materials and Methods

### 2.1. Ficus benghalensis Bark Extract Preparation

Plant material was procured from a commercial online platform and verified by the Department of Botany and Microbiology, College of Sciences, King Saud University, Riyadh. The bark of *Ficus benghalensis* was cut into small pieces, washed, dried, and then pulverized. Twenty grams of bark powder was mixed with 200 mL of deionized H_2_O and stirred at 40 °C for 2 h. The concoctions were filtered, and the filtrate was used as an aqueous extract.

### 2.2. Fabrication of ZnO-NPs

A total of 100 mL of Zinc chloride (0.1 mol L^−1^) was agitated for 30 min at 60 °C, and the alkaline condition was maintained. To this mixture, 10 mL of bark extract was added, stirred, and maintained at 90 °C for 15 min. The white precipitate was obtained and further subjected to 3000× *g* for 10–15 min. The pellet was collected and dried in a muffle furnace at 250 °C for 3 h [12]. The synthesized nanoparticles were re-dispersed in deionized water and used for characterization and biological experiments.

### 2.3. Characterization of ZnO-NPs

UV–Vis spectrophotometry: The reduced zinc ions were confirmed by UV–Vis spectral analysis by taking 1 mL of the sample and comparing it with water as a blank. UV–Vis Spectral analysis was carried out in a Shimadzu UV–Vis Spectrophotometer (Shimadzu Corporation, Kyoto, Japan).

FTIR analysis: The sample was mixed with KCl and analyzed in a Shimadzu FTIR Prestige 21 (Shimadzu Corporation, Kyoto, Japan) in the range of 4000–400 cm^−1^ at a resolution of 4 cm^−1^. The sample disk was placed and analyzed for nanoparticles.

SEM-EDAX analysis: The nanoparticle suspension was analyzed using a LEO 1355 VP (LEO Electron Microscopy Ltd., Cambridge, UK) equipped with an energy-dispersive X-ray detector. A drop of suspension was placed in an electric stub and allowed to evaporate without any traces of water in it.

Zeta potential and PSA: Zeta potential analysis was performed to analyze the reduction and stability of ZnO-NPs with particle size distribution using the Malvern Zetasizer (Malvern Panalytical Ltd., Malvern, UK).

XRD analysis: The crystallite quality of ZnO-NPs was assessed by employing a Rigaku Miniflex 600 X-ray diffractometer (Rigaku Corporation, Tokyo, Japan) with a high-power Cu K source operating at 40 kV/15 mA.

### 2.4. In Vitro Studies

#### 2.4.1. Cell Line

Osteosarcoma cell line Saos-2 was procured from ATCC (Manassas, VA, USA). The cells were cultured in 10% DMEM with 2% Pen/Strep antibiotic and 10% fetal bovine serum (FBS) (Sigma-Aldrich, St. Louis, MO, USA), followed by incubation in a humidified 5% CO_2_ at 37 °C. Every 2–3 days, the new DMEM was replaced until the desired confluency was attained.

#### 2.4.2. MTT Assay

Initially, the MTT assay was used to validate the anticancer capability of ZnO-NPs on Saos-2 cells [19]. Additionally, 1 × 10^4^ cells per well were seeded in each well of 96-well plates. Once they reached a confluency of 80–90%, the cells were subjected to various dosages of ZnO-NPs, such as 0.2, 0.4, 0.8, 1.6, 3.125, 6.25, 12.5, 25, 50, 75, and 100 μg mL^−1^ for 24 h. Then, 15 µL of MTT solution was added to each well in the dark and incubated for 4 h. The purple complex dissolved with 200 µL of DMSO. The microtiter plate reader (BioTek Instruments, Inc., Winooski, VT, USA) was utilized to read the plate at 490 and 630 nm, and the percentage of inhibition was calculated. The MTT assay was also performed on the *Ficus benghalensis* bark extract at dosages of 100, 50, 25, 12.5, 6.25, 3.125, 1.6, 0.8, and 0.4 μg mL^−1^ towards Saos-2 cells. Furthermore, the assay was also applied to the standard chemotherapeutic agent Doxorubicin and the normal cell line BEAS-2B (human bronchial epithelial cell line).

#### 2.4.3. Trypan Blue Exclusion Assay

The trypan blue exclusion assay was performed to check the viability of treated cells with the intent of validating the cytotoxicity of ZnO-NPs [20]. Saos-2 cells were subjected to 50, 75, and 100 μg mL^−1^ of ZnO-NPs for 24 h after attaining 80% confluency. Subsequently, treated and untreated cells were exposed to a PBS wash. Cells were trypsinized and resuspended in fresh DMEM and further stained with trypan blue dye (0.4%). Using a hemocytometer, live and dead cells were manually counted. The percentage of viability was calculated: Viability (%) = No. of viable cells/Total No. of cells × 100.

#### 2.4.4. Morphological Analysis

The morphometric alterations of treated cells were studied further to verify the anticancer potential of ZnO-NPs at various dosages of ZnO-NPs (50, 75, and 100 μg mL^−1^) for 24 h. After discarding the existing DMEM, treated and control cells were subjected to a PBS wash. The morphometric deformations in the treated groups were observed, and images were taken using a Phase Contrast microscope (Olympus, Tokyo, Japan) [21].

#### 2.4.5. DAPI Staining

Using the DAPI staining method, the apoptotic capability of ZnO-NPs towards Saos-2 cells was investigated [22]; 1 × 10^5^ cells of appropriate count were seeded. Grown cells were exposed to 50, 75, and 100 μg mL^−1^ for 24 h. Subsequently, the cells were subjected to washing in cooled PBS and fixed in chilled methanol for 10 min. After permeabilizing the cells with 4% formaldehyde, they were stained with DAPI dye (0.5 μg mL^−1^) and rinsed for further observation. Finally, the images were captured utilizing a fluorescence microscope (Nikon, Tokyo, Japan).

### 2.5. Oxidative Stress Biomarkers Estimation

Intracellular reactive oxygen species (ROS), nitric oxide (NO), and lipid peroxidation (LPO) were measured in Saos-2 cells after treatment with ZnO-NPs (50, 75, and 100 μg mL^−1^) in the absence and presence of N-acetylcysteine (NAC) at a concentration of 10 mM. The cells were pretreated with NAC for 2 h. For ROS analysis, 1 × 10^4^ cells per well were seeded in 96-well plates, treated for 24 h, followed by washing with PBS, and subjected to incubation with 0.1% nitro blue tetrazolium (NBT) for 1 h. The reaction product was solubilized in KOH and DMSO, and absorbance was read at 630 nm. NO levels were determined by the Griess assay using culture supernatants mixed with sulfanilamide and N-(1-naphthyl)ethylenediamine dihydrochloride (NEDA), followed by absorbance measurement at 540 nm. LPO was evaluated by the TBARS method using cell lysates reacted with sodium dodecyl sulfate, acetic acid, and thiobarbituric acid, heated at 95 °C for 1 h, and read at 532 nm. NO and LPO were expressed as μM and μM malondialdehyde equivalents, respectively.

### 2.6. Antioxidant Markers

#### 2.6.1. Assay for Catalase

The catalase assay was carried out as suggested in [23] with minor changes. The cells were subjected to different IC_50_ dosages of ZnO-NPs for 24 h. First, 1 mL of freshly prepared substrate (13.3 mL of 30% of H_2_O_2_ was added with 6.7 mL of 100 mM phosphate buffer (pH 7.4)) was added to 0.5 mL of treated and control cell suspension. Then, it was incubated in dark conditions at room temperature for 2 min. Then, 1 mL of 32.4 mM ammonium molybdate was added to the complex to halt the enzymatic reaction. The resulting yellow-colored complex was read at 405 nm. The catalase activity was expressed in KU/L.

#### 2.6.2. Assay for SOD

The activity of SOD was assessed by performing the procedure of [24] with slight variations. The cells were treated with ZnO-NPs (50, 75, and 100 μg mL^−1^) for 24 h. A total of 200 µL of the treated and control cell suspension was added to 200 µL of NBT (0.08 mM), 400 µL of NADH (0.25 mM), and 200 µL of PMS. The complex was kept in the dark for 10 min at room temperature. The absorbance was recorded at 560 nm. The release of SOD was determined.

SOD release (%) = A0 − A1/A0 × 100

where A1 = optical density of the sample and A0 = optical density of the control.

#### 2.6.3. Assay for GSH

The reduced glutathione assay was performed by the method described in [25] with few modifications. The treated and control cells were subjected to trypsinization and centrifugation. The resulting pellets were homogenized using 0.1 mol L^−1^ potassium phosphate buffer (pH 7.4). An equal amount of 4% sulfosalicylic acid was added with 0.5 mL of homogenate and kept for incubation at 4 °C for 1 h. The complex was subjected to centrifugation, and 0.03 mL of supernatant was taken separately. To that, 0.9 mL of 0.1 mol L^−1^ Potassium phosphate buffer (pH 7.4), and 0.066 mL of DTNB were added. The absorbance was read at 412 nm. The reduced glutathione was denoted as mM.

#### 2.6.4. Analysis of Caspase Activity

The quantification of caspase-8 further confirmed the apoptotic potential of ZnO-NPs towards Saos-2 cells. The colorimetric test kits’ (Elab Science, Houston, TX, USA) guidelines were followed for experimenting with the activity of caspase-8. In a 96-well plate, 1 × 10^4^ cells per well were seeded. Once they reached 80% confluency, they were exposed to 50, 75, and 100 μg mL^−1^ ZnO-NPs for 24 h. Using ice-cold phosphate-buffered saline, the cell lysate was prepared. To 30 µL of cell lysate, 20 µL of caspase-8 substrate (Ac-IETD-pNA) and 150 µL of reaction buffer were added and incubated for 15 min. The reaction mixture’s fluorescence was measured at 405 nm by employing an ELISA reader (ELX-800n, Biotek, Shoreline, WA, USA).

#### 2.6.5. Flow Cytometry Analysis

Saos-2 cells were harvested in 6-well plates and treated with 50, 75, and 100 μg mL^−1^ of ZnO-NPs for 24 h. The cells were harvested, washed with PBS, and fixed in ice-cold 70% ethanol. The fixed cells were then stained with 20 μg/mL PI in the presence of RNase A and finally subjected to incubation in a dark environment before analysis. Flow cytometric acquisition was performed using a flow cytometer, and at least 10,000 events were collected per sample. Cell-cycle distribution was evaluated from DNA content histograms, and the percentages of cells in the G0/G1, S, and G2/M phases were calculated. The analysis was performed using FlowJo software version 11 (FlowJo LLC, Ashland, OR, USA.) with manual sequential gating, rather than a cell-cycle deconvolution algorithm. Additionally, analysis followed a standard sequential gating strategy commonly used for DNA-content analysis, consisting of (i) exclusion of debris based on forward- and side-scatter characteristics, (ii) rigorous doublet discrimination to retain true single cells, and (iii) identification of DNA-content populations using PI fluorescence intensity. The same gating hierarchy and gate positions were applied consistently to all experimental groups to enable direct comparison between treatments [26].

#### 2.6.6. Gene Expression Studies of Bcl-2, Bax, and p53

The expression of anti-apoptotic marker Bcl-2 and apoptotic markers such as Bax and p53 was analyzed using qPCR. Briefly, total RNA was isolated from the treated and control groups by employing TRIZOL RNA extraction solution (Takara, Cat. No: 9108, Kusatsu City, Japan). Gene expression analysis was carried out by performing PCR for amplification of β-actin (Forward 5′GCAGATGTGGATCAGCAAGC 3′ and Reverse 5′ GCAGCTCAGTAACAGTCCGC 3′), used as an internal reference: Bcl-2 (Forward 5′ TACCTGAACCGGCACCTG 3′ and Reverse 5′ GCCGTACAGTTCCACAAAGG 3′), Bax (Forward 5′ CCTGCTTCTTTCTTCATCGG 3′ and Reverse 5′AGGTGCCTGGACTCTTGGGT 3′), and p53 (Forward 5′ CTCTGACTGTACCACCATGG 3′ and Reverse 5′ CCGTCCCAGTAGATTACCAC 3′). The reaction was conducted using TB green premix Ex Tag II master mix (Takara, Cat. No. RR820A). A total of 2 μL of cDNA (5 ng/μL), 12.5 μL of TB Green Master mix, 1 μL of forward primer, 1 μL of reverse primer, and 8.5 μL of RNase-free H_2_O make up the reaction mixture, resulting in a total volume of 20 μL. The initial condition was set at 95 °C for 30 min. The denaturation was set at 95 °C for 15 s. The annealing and extension were set at 54.2 °C for 30 s. The reactions were run for 40 cycles. The experiments were performed in triplicate in a 96-well format on the Bio-Rad CFX96 Real-Time system (Bio-Rad Laboratories, Hercules, CA, USA). The relative expression of mRNA was determined using the 2^−∆∆Ct^ method.

### 2.7. Statistical Analysis

GraphPad Prism (version 8.1) was utilized for statistical analysis. The experiments were carried out in triplicate, and outcomes were evaluated using one-way ANOVA. Dunnett’s multiple comparison tests were applied to compare the treated and control groups. Data are represented as Mean ± SD. *p* < 0.0001 was considered significant.

## 3. Results

### 3.1. Characterization of ZnO-NPs

**Figure 1:**
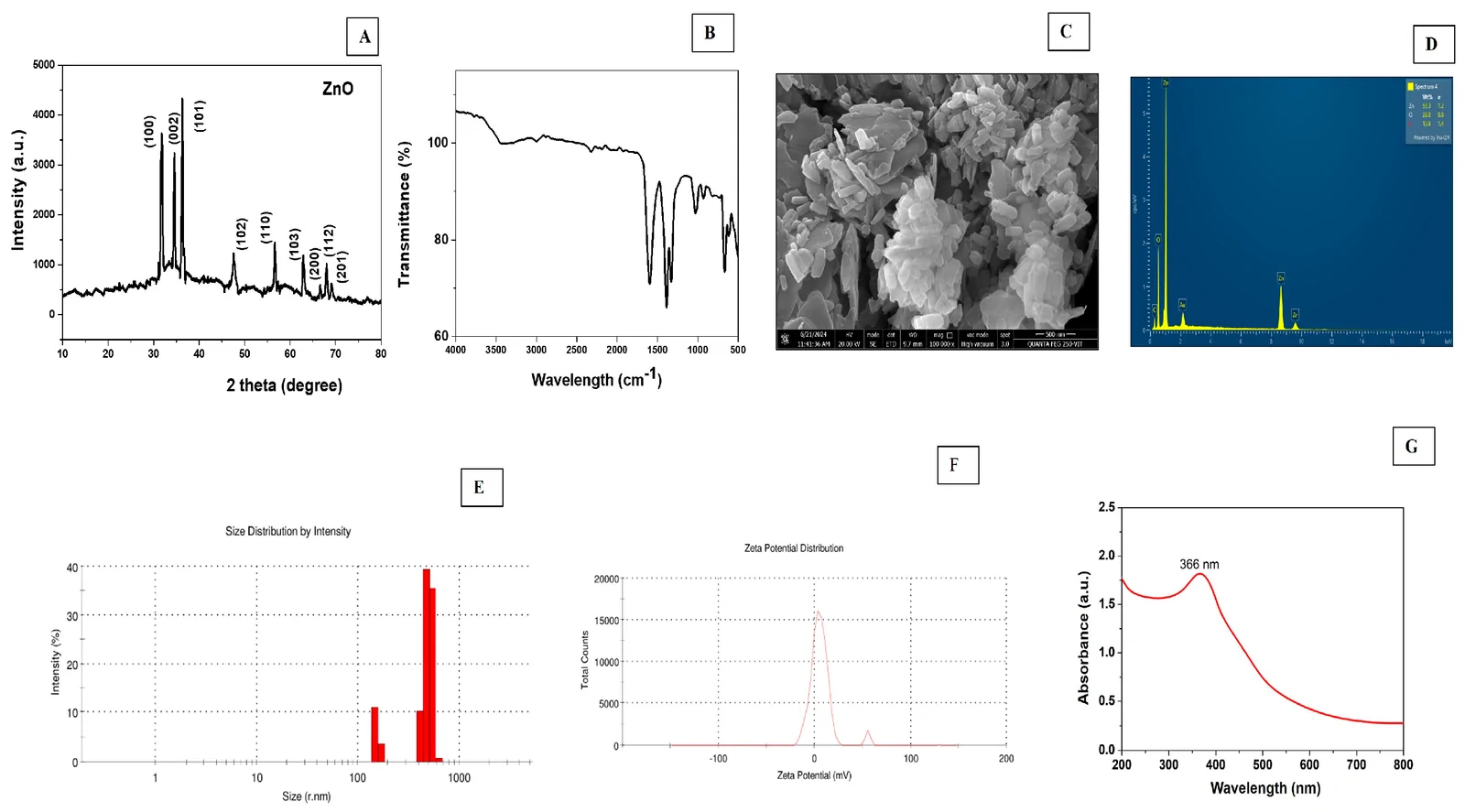
Physicochemical properties of biogenic ZnO nanoparticles (ZnO-NPs). (**A**) X-ray diffraction (XRD) pattern of ZnO-NPs establishes the crystalline structure and phase purity. (**B**) Fourier-transform infrared spectroscopy (FTIR) spectrum demonstrating the functional groups associated with phytochemical capping agents and Zn–O bond vibrations. (**C**) Field-emission scanning electron microscopy (FESEM) micrograph illustrating the surface morphology and rod-like structure of ZnO-NPs. (**D**) Energy-dispersive X-ray analysis (EDX) spectrum and elemental composition representing the weight and atomic percentages of constituent elements in ZnO-NPs. (**E**) Particle size distribution of ZnO-NPs, indicating the average particle size and distribution range. (**F**) Zeta potential distribution of ZnO-NPs representing the surface charge characteristics. The dominant peak corresponds to the major nanoparticle population, whereas minor peaks denote subpopulations with different surface charge distributions. (**G**) UV–visible absorption spectrum of ZnO-NPs demonstrating a characteristic absorption peak at approximately 366 nm, establishing the formation of ZnO nanoparticles.

In XRD analysis, the corresponding angle and lattice planes are as follows, 31.73° (100), 34.50° (002), 36.37° (101), 47.65° (102), 56.81° (110), 62.94° (103), 66.58° (200), 67.82° (112), and 69.21° (201). The average crystallite size determined by the Debye–Scherer formula is 28 nm (Figure 1A). The FTIR spectrum of ZnO-NPs exhibited distinct peaks at 3442, 2998, 2319, 1601, 1366, 1314, 1026, 922, 647, and 635 cm^−1^. The broad peak was at 3442 cm^−1^ owing to O-H stretching vibrations. Peaks at 2998, 1366, and 922 cm^−1^ correspond to C–H stretching and bending vibrations. The 2319 cm^−1^ peak is linked to CO_2_ or CO stretching vibrations, while the 1601 cm^−1^ peak is associated with O-H bending or C=O stretching vibrations. The peak at 1026 cm^−1^ is attributed to C–O stretching vibrations. The peaks at 647 and 635 cm^−1^ are characteristic of Zn-O stretching vibrations (Figure 1B), and FESEM demonstrated the morphology of ZnO-NPs (Figure 1C). The surface morphology was observed as a rod-like structure and unevenly aggregated. The EDAX spectrum analysis confirmed the presence of Zn and O elements in the synthesized ZnO-NPs (Figure 1D). The surface Plasmon resonance of ZnO-NPs showed an absorption peak, which was observed and identified as zinc, oxygen, and carbon in the atomic percentages of 21, 45, and 33, respectively. The weight percentages of zinc, oxygen, and carbon are 55, 28, and 15, respectively. The particle size distribution of phytogenically fabricated ZnO-NPs was found to be 502.5 nm, as depicted in Figure 1E. The zeta potential distribution represented a predominant peak centered at approximately +5 to +10 mV, associated with a minor population of particles demonstrating higher positive charge around +55–60 mV, as shown in Figure 1F. The UV–Vis absorption spectrum of ZnO-NPs demonstrated a distinct peak at 366 nm (Figure 1G).

### 3.2. MTT Assay

**Figure 2:**
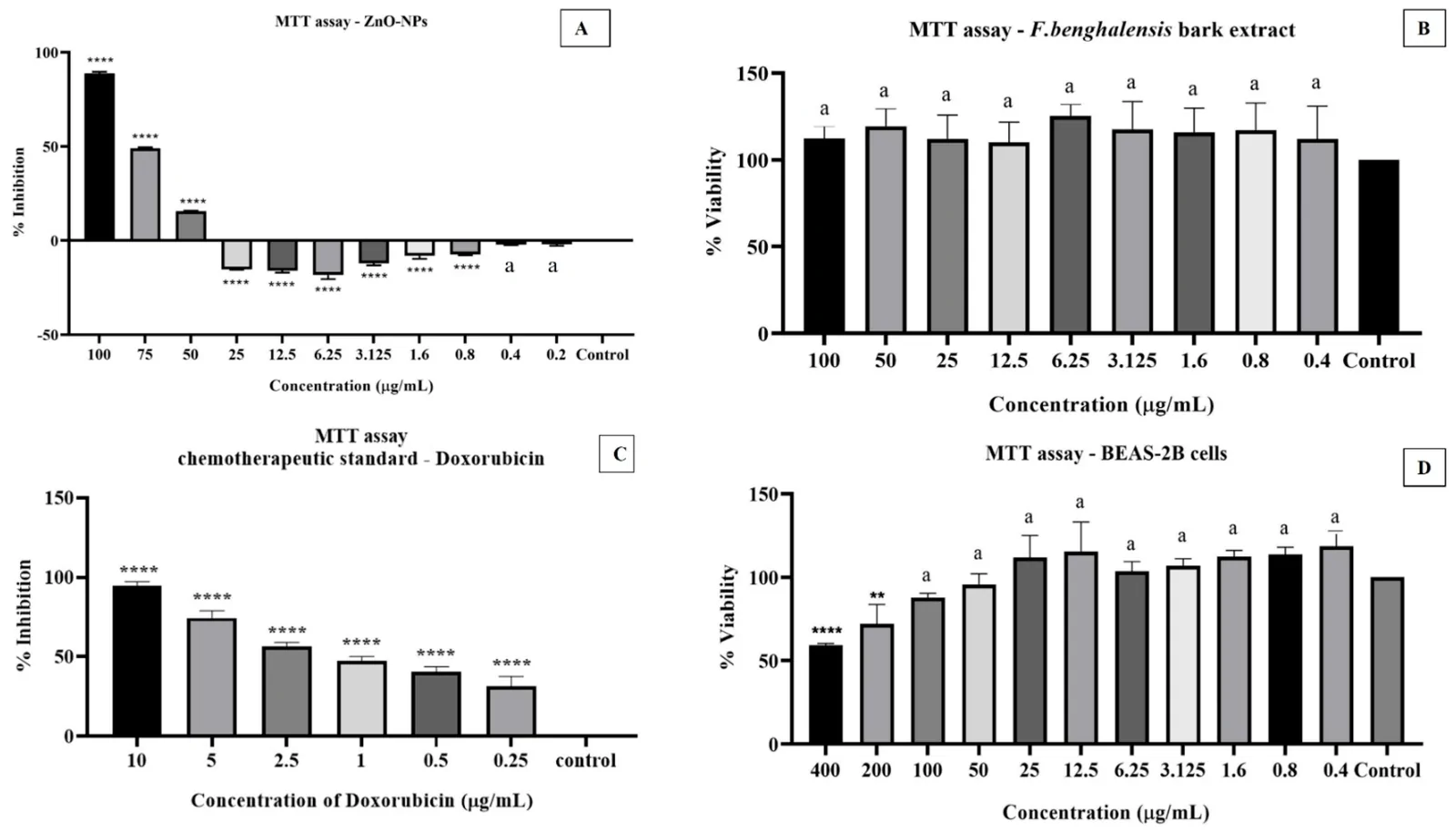
MTT assay: (**A**) Cytotoxic effects of biogenic ZnO-NPs (0.2–100 μg mL^−1^) on Saos-2 cells. (**B**) Cytotoxic effects of *Ficus benghalensis* bark extract (0.4–400 μg mL^−1^) on Saos-2 cells. (**C**) Cytotoxic effects of doxorubicin (positive control) on Saos-2 cells. (**D**) Cytotoxic effects of ZnO-NPs on BEAS-2B human bronchial epithelial cells. Data are presented as mean ± SD (*n* = 3). Statistical analysis was performed using one-way ANOVA followed by Dunnett’s multiple comparisons test. Asterisks indicate significant differences compared with the untreated control (**p < 0.01, ****p < 0.0001). ‘a’ represents non-significance.

The cytotoxic impact of ZnO-NPs mediated by *Ficus benghalensis* was tested on Saos-2 cells using the MTT reagent. First, 100 μg mL^−1^ (high dose) exhibited 89.36% inhibition. IC_50_ was observed at the dosage of 75 μg mL^−1^, which exhibited 49.36% inhibition, and 50 μg mL^−1^ showed 15.74%. No inhibitory effect was observed up to 25 μg mL^−1^ (Figure 2A). The cytotoxic potential of plain *Ficus benghalensis* bark extract was evaluated against Saos-2 osteosarcoma cells, demonstrating no cytotoxic effects up to 100 μg mL^−1^ (Figure 2B). Further, doxorubicin treatment demonstrated a dosage-dependent increase in cell suppression, with the highest inhibition observed at 10 μg mL^−1^, suggesting the susceptibility of Saos-2 cells to the chemotherapeutic drug (Figure 2C). Additionally, evaluation of BEAS-2B cells revealed the biocompatibility of ZnO-NPs towards a normal cell line (Figure 2C). A significant drop in viability was noticed only at the highest concentrations, with viability decreasing to approximately 70% and 60% at 200 and 400 µg mL^−1^.

### 3.3. Trypan Blue Exclusion Assay

**Figure 3:**
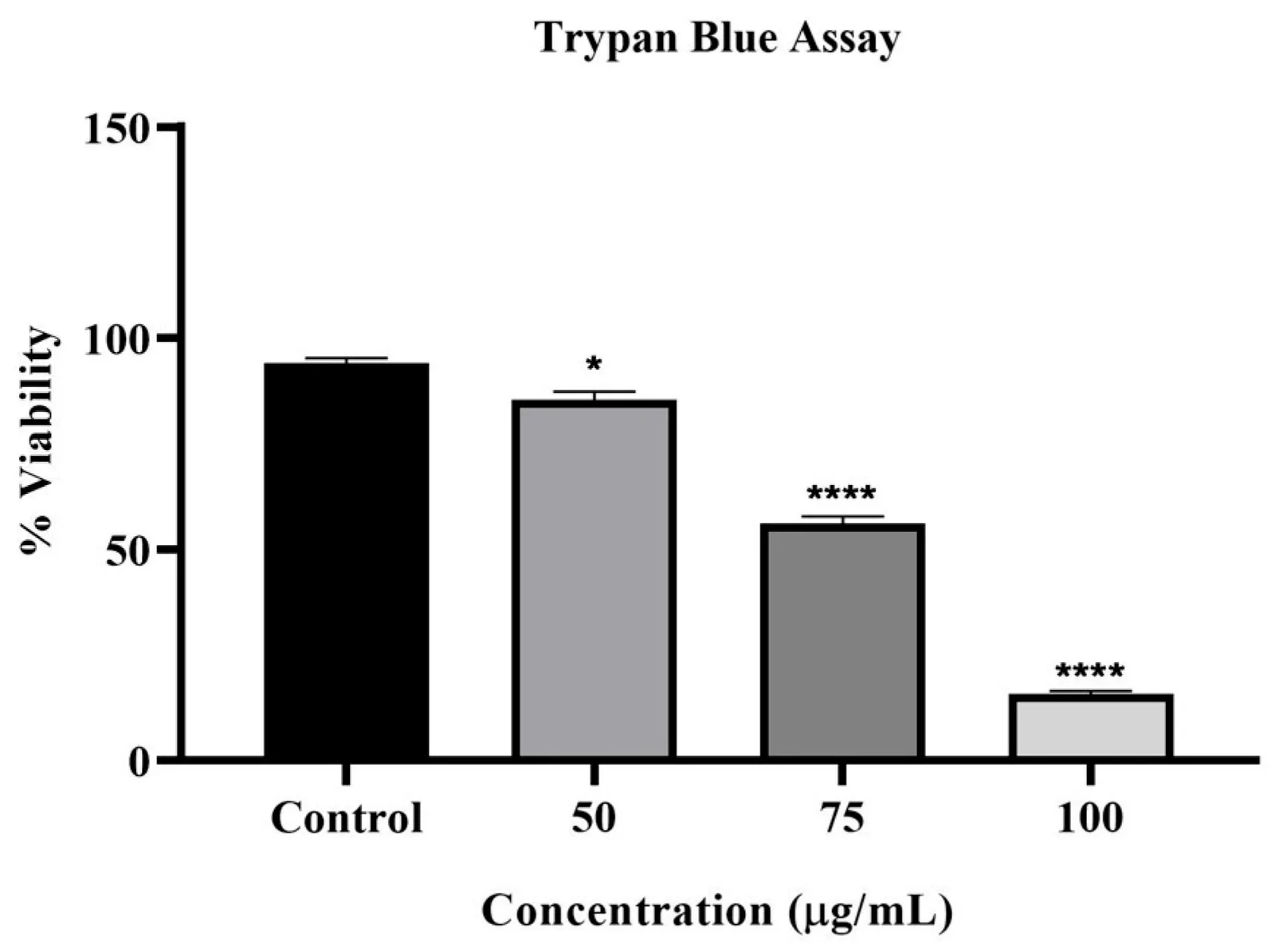
Assessment of cell viability by trypan blue exclusion assay. Viability of Saos-2 cells following exposure to ZnO-NPs at different concentrations. Data are denoted as mean ± SD (*n* = 3). Statistical significance is indicated as * *p* < 0.05, **** *p* < 0.0001. Error bars represent standard deviation.

The dosages such as 50, 75, and 100 μg mL^−1^ were selected for the viability assay based on the MTT data. It was evident from Figure 3 that the number of live cells was reduced notably at 100 μg mL^−1^, which exhibited 15.79% viability. Additionally, 85.41% and 56.11% viability were noticed at 50 and 75 μg mL^−1^, respectively. The trypan blue exclusion assay findings were correlated with the MTT assay, proving that ZnO-NPs exert anticancer effects on Saos-2 cells.

### 3.4. Morphometric Analysis

**Figure 4:**
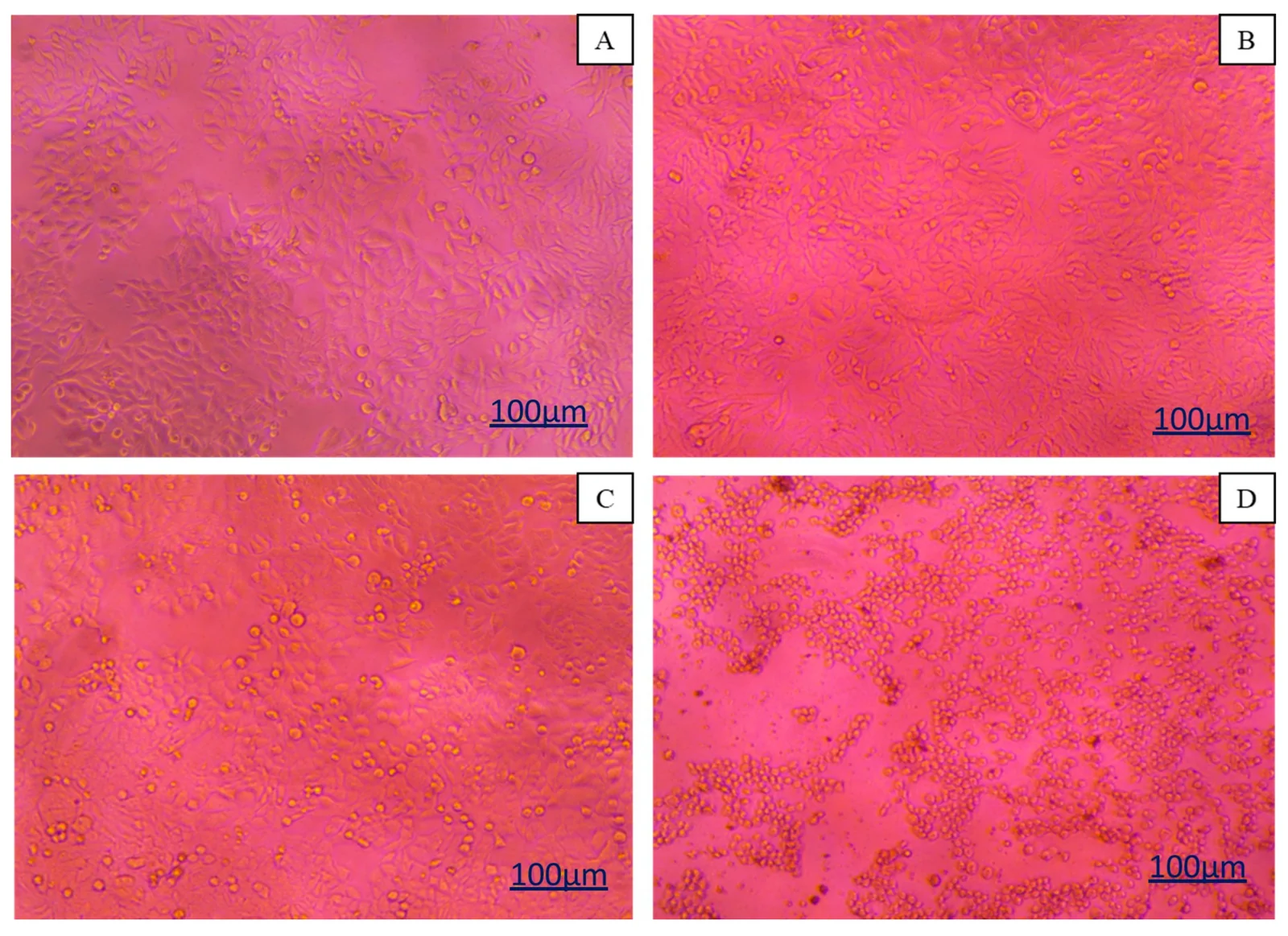
Morphological alterations in Saos-2 cells after ZnO-NP treatment. Representative microscopic images of untreated and ZnO-NPs-treated Saos-2 cells. (**A**) untreated control, (**B**) 50 μg mL^−1^, (**C**) 75 μg mL^−1^, and (**D**) 100 μg mL^−1^. Untreated cells exhibited normal morphology and intact cellular architecture, whereas treated cells demonstrated concentration-dependent morphological alterations, including cell shrinkage and loss of cellular integrity, which are signs of cytotoxic effects.

An additional crucial factor for evaluating the anti-proliferation effect of ZnO-NPs (50, 75, and μg mL^−1^) on Saos-2 cells is the morphological observation of the treated cells. The treated cells displayed more detached cells than the control cells. Furthermore, there are notable changes in the morphology, such as cell shrinkage and rupturing, cell blebbing, and disoriented cells, as shown in Figure 4.

### 3.5. DAPI Staining

**Figure 5:**
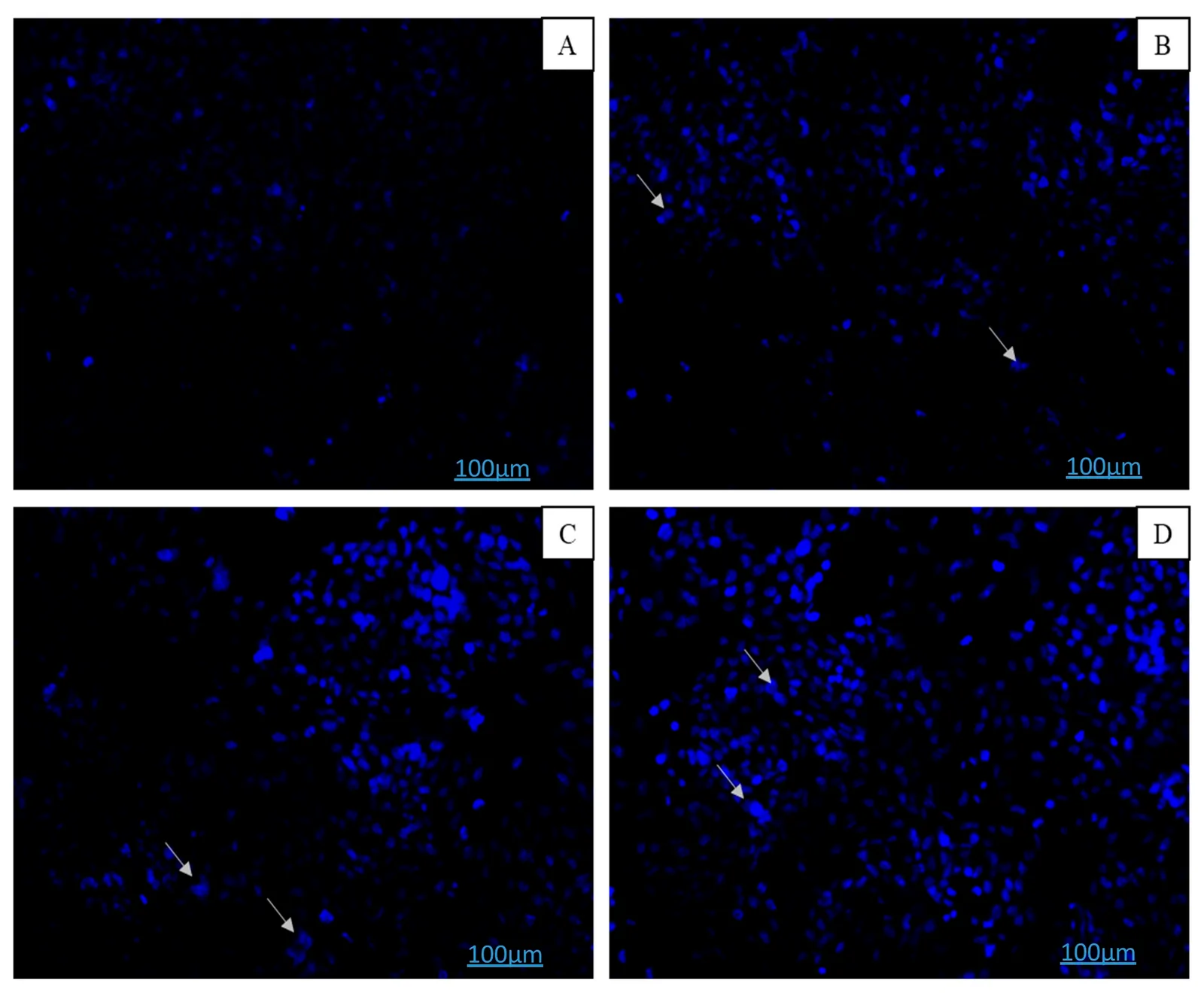
Nuclear morphological modifications in Saos-2 cells by DAPI staining. Representative fluorescence micrographs of control and ZnO-NPs-treated Saos-2 cells stained with DAPI. (**A**) Untreated control, (**B**) 50 μg mL^−1^, (**C**) 75 μg mL^−1^, and (**D**) 100 μg mL^−1^. Control cells exhibited uniformly stained nuclei with intact nuclear morphology, whereas ZnO-NP-treated cells demonstrated concentration-dependent nuclear condensation and fragmentation (arrows are marked).

DAPI staining was performed to examine the nuclear deformities of Saos-2 cells at 50, 75, and 100 μg mL ^1^ dosages. The staining images, along with apoptotic cells, were marked with arrows and depicted in Figure 5. The outcomes demonstrated an increase in blue fluorescence cells in a dose–response manner, indicating the antiproliferative activity of ZnO-NPs. The treated cells demonstrated fragmentation of chromatin and condensed nuclei. Meanwhile, untreated cells showed a normal structure without any deformations.

### 3.6. Oxidative Stress Biomarkers Estimation

**Figure 6:**
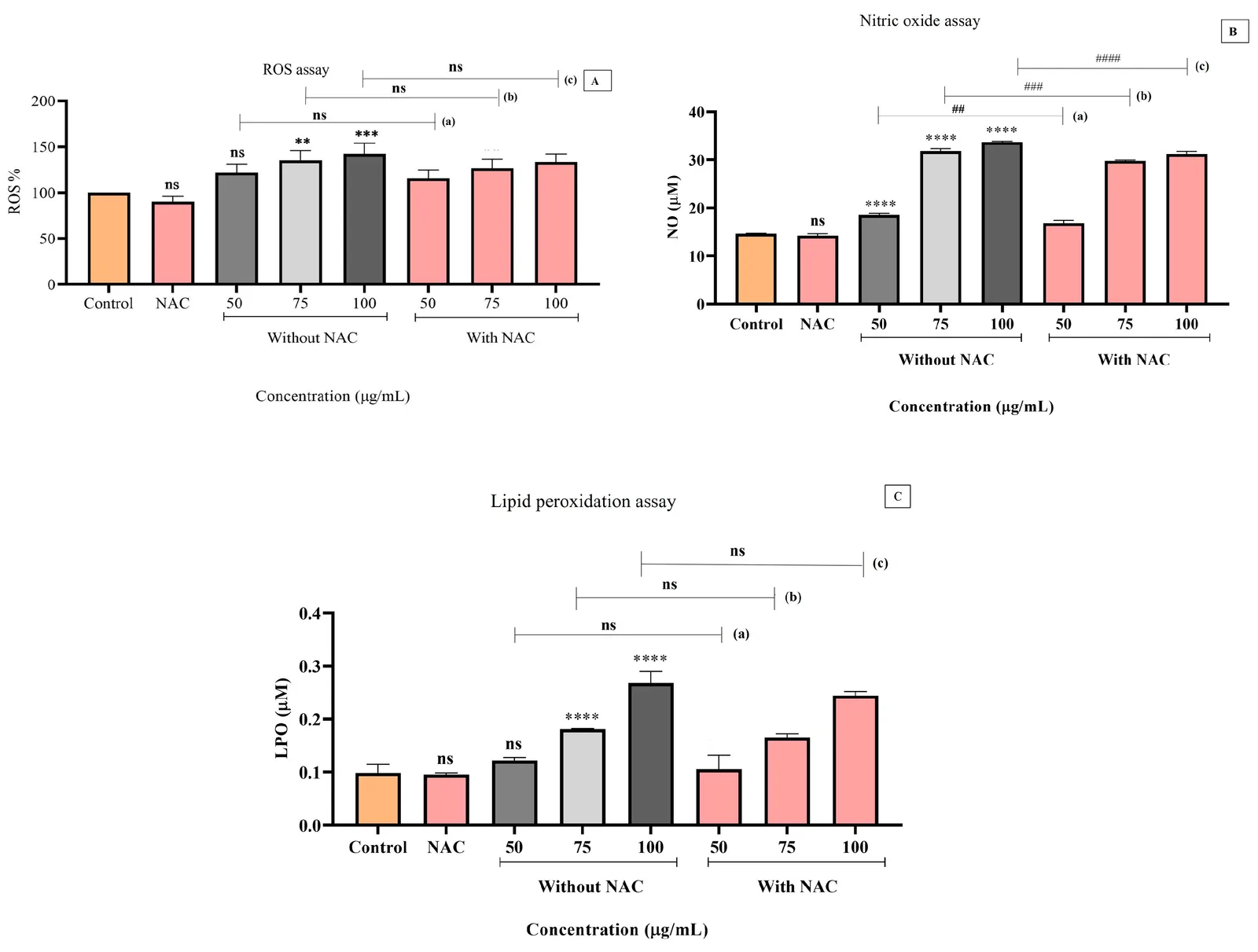
Effect of N-acetylcysteine (NAC) on ZnO-NPs in Saos-2 osteosarcoma cells. (**A**) Intracellular reactive oxygen species (ROS) levels, (**B**) nitric oxide (NO) production, and (**C**) lipid peroxidation (LPO) levels in Saos-2 cells following treatment with biogenic ZnO-NPs (50, 75, and 100 μg mL^−1^) in the absence and presence of NAC. ZnO-NPs exposure resulted in a dosage-dependent increase in ROS generation, NO production, and LPO levels compared with untreated control cells. Pre-treatment with NAC significantly attenuated these oxidative stress markers, indicating the involvement of ROS-dependent mechanisms. Data are presented as mean ± SD (*n* = 3). Statistical significance was analyzed using one-way ANOVA followed by Dunnett’s multiple comparison test. ** *p* < 0.01, *** *p* < 0.001, **** *p* < 0.0001. ## *p* < 0.01, ### *p*< 0.001, #### *p* < 0.0001. #—indicates the statistical comparison between the without NAC groups towards with NAC group. (a) Represents the comparison between 50 μg mL^−1^ of the without NAC group and 50 μg mL^−1^ of the with NAC group. (b) Represents the comparison between 75 μg mL^−1^ of the without NAC group and 75 μg mL^−1^ of the with NAC group. (c) Represents the comparison between 100 μg mL^−1^ of without NAC group towards 100 μg mL^−1^ of with NAC group. ns—non-significant.

The study of oxidative biomarkers further supports the cytotoxic effects of biogenic ZnO-NPs in Saos-2 cells. The findings demonstrated that exposure to ZnO-NPs resulted in elevated levels of ROS in different dosages compared with untreated control cells. The NAC-alone-treated group did not exhibit ROS generation (Figure 6A). However, pre-treatment with NAC reduced ROS accumulation in cells exposed to ZnO-NPs. Although ROS levels remained slightly elevated compared with untreated controls, the marked attenuation observed in NAC-pretreated groups indicates that ZnO-NPs-induced oxidative stress is predominantly mediated through intracellular ROS generation. In the ROS assay, the control group was compared with NAC, which exhibited 90.09 ± 6.32 (*p* = 0.8611). The dosages such as 50 μg mL^−1^ showed 121.69 ± 9.41 (*p* = 0.1188), 75 μg mL^−1^ demonstrated 135.26 ± 10.66 (*p* = 0.0034), and 100 μg mL^−1^ exhibited 142.49 ± 11.61 (*p* = 0.0005). Additionally, 50 μg mL^−1^ without NAC was compared with 50 μg mL^−1^ of with NAC, which showed non-significance with *p* = 0.9887. Similarly, 75 μg mL^−1^ of without NAC was compared with 75 μg mL^−1^ of with NAC showed non-significance with *p* = 0.9285, and 100 μg mL^−1^ showed *p* = 0.9034. In the NO assay, ZnO-NPs treatment significantly increased nitric oxide (NO) production in Saos-2 cells in a dosage-dependent manner compared with the control group, suggesting enhanced nitrosative stress following nanoparticle exposure (Figure 6B). The control group was compared with NAC, which exhibited non-significance with *p* = 0.8996. No significant difference was observed between the control and NAC-alone groups. However, NAC pre-treatment markedly reduced ZnO-NPs-induced NO generation at all tested dosages. This attenuation suggests that ROS production contributes to NO overproduction and the associated oxidative damage in ZnO-NPs-treated cells. LPO level was also elevated in ZnO-NPs-treated cells compared with the control group, indicating increased oxidative damage to membrane lipids (Figure 6C). NAC alone did not significantly affect the LPO level, whereas NAC pre-treatment significantly suppressed ZnO-NPs-induced lipid peroxidation. The control was compared with NAC, which exhibited non-significance with *p* = ≥ 0.999. The dosage 50 μg mL^−1^ exhibited *p* = 0.4966. Furthermore, 50 μg mL^−1^ of without NAC was compared with 50 μg mL^−1^ of with NAC, which showed non-significance with *p* = 0.8370. Similarly, 75 μg mL^−1^ of without NAC was compared with 75 μg mL^−1^ of with NAC, which showed non-significance with *p* = 0.8471, and 100 μg mL^−1^ showed *p* = 0.5095.

### 3.7. Antioxidant Markers

#### 3.7.1. Assay for Catalase

**Figure 7:**
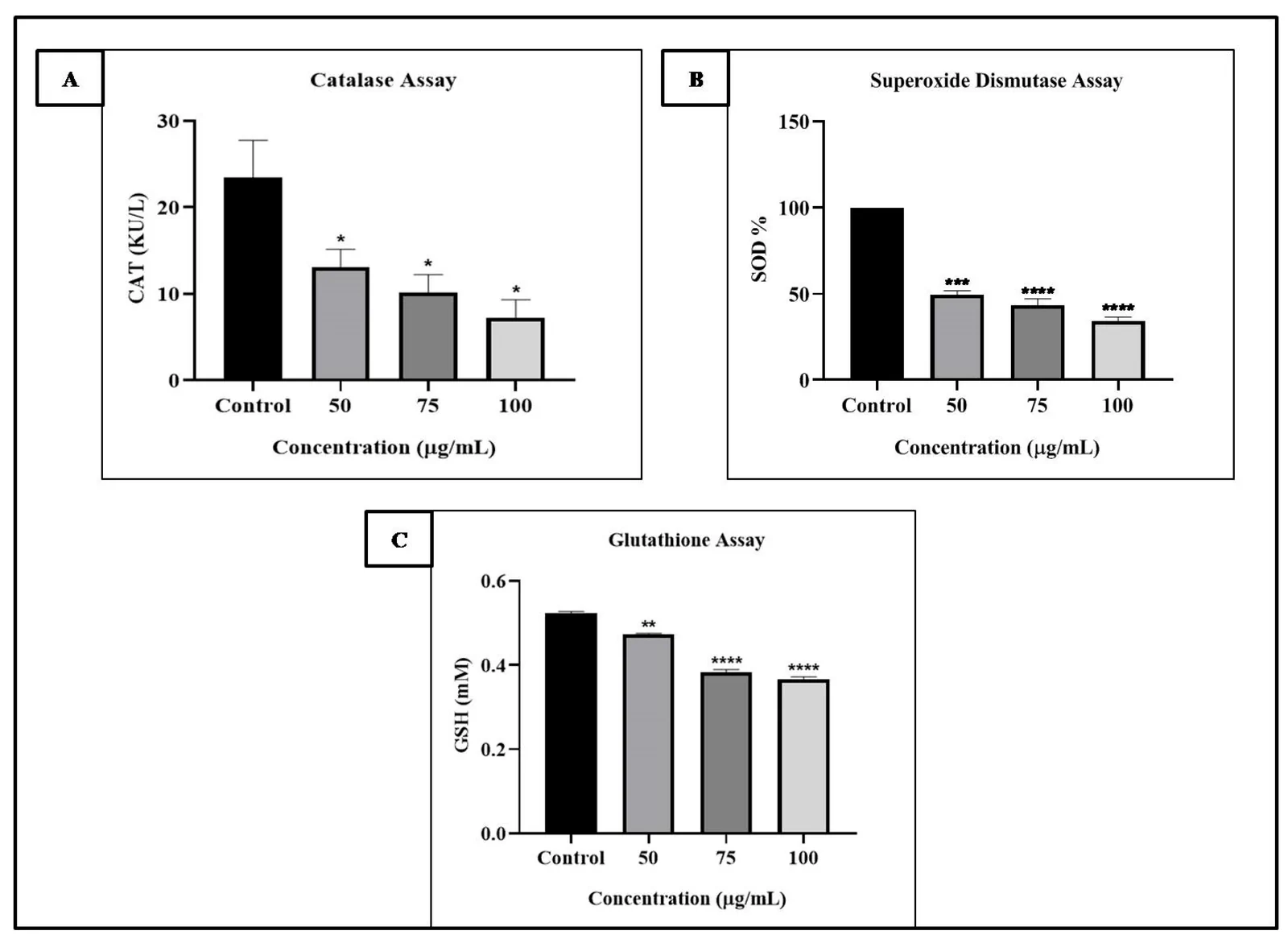
Effect of ZnO-NPs on intracellular antioxidant defense systems in Saos-2 cells. (**A**) Catalase (CAT) activity, (**B**) superoxide dismutase (SOD) activity, and (**C**) reduced glutathione (GSH) levels after ZnO-NP treatment. Data are denoted as mean ± SD (*n* = 3). Statistical significance was determined using one-way ANOVA followed by Dunnett’s multiple comparison test. * *p* < 0.05, ** *p* < 0.01, *** *p* < 0.001, **** *p* < 0.0001.

The catalase activity of Saos-2 cells treated with ZnO-NPs reduced considerably with increasing dosages (Figure 7A). The control cells showed 23.26 ± 4.07 KU/L, and dosages such as 50 μg mL^−1^ showed 15.26 ± 2.18 (*p* = 0.0470), 75 μg mL^−1^ demonstrated 10.17 ± 1.45 KU/L (*p* = 0.0207), and 100 μg mL^−1^ exhibited 7.85 ± 2.03 KU/L (*p* = 0.0103). The findings of catalase activity are statistically significant with *p* < 0.05.

#### 3.7.2. Assay for SOD

A similar pattern of outcomes was observed in the SOD depicted in Figure 7B. The control cells exhibited 100% of the SOD level. On the other hand, the level of SOD decreased gradually in the treated group, which had 49.40 ± 2.40, 43.46 ± 3.60, and 34.12 ± 2.40 for dosages of 50, 75, and 100 μg mL^−1^, respectively. The results were analyzed using one-way ANOVA. Dunnett’s multiple comparison test demonstrated a significant difference between the control and treated groups (*p* < 0.0001).

#### 3.7.3. Assay for GSH

In the GSH assay, a reduction in levels of glutathione was observed with an increase in the dose compared to the control (Figure 7C). The control demonstrated 0.52 ± 0.003 µM. The doses of 50, 75, and 100 μg mL^−1^ showed 0.47 ± 0.001, 0.38 ± 0.005, and 0.36 ± 0.005 µM, respectively. The control group had 0.52 ± 0.03 µM. A one-way ANOVA test demonstrated that the results are highly significant with *p* < 0.0001. It was clear from the assays that ZnO-NPs exert remarkable anticancerous activity on Saos-2 cells.

### 3.8. Analysis of Caspase-8 Activity

**Figure 8:**
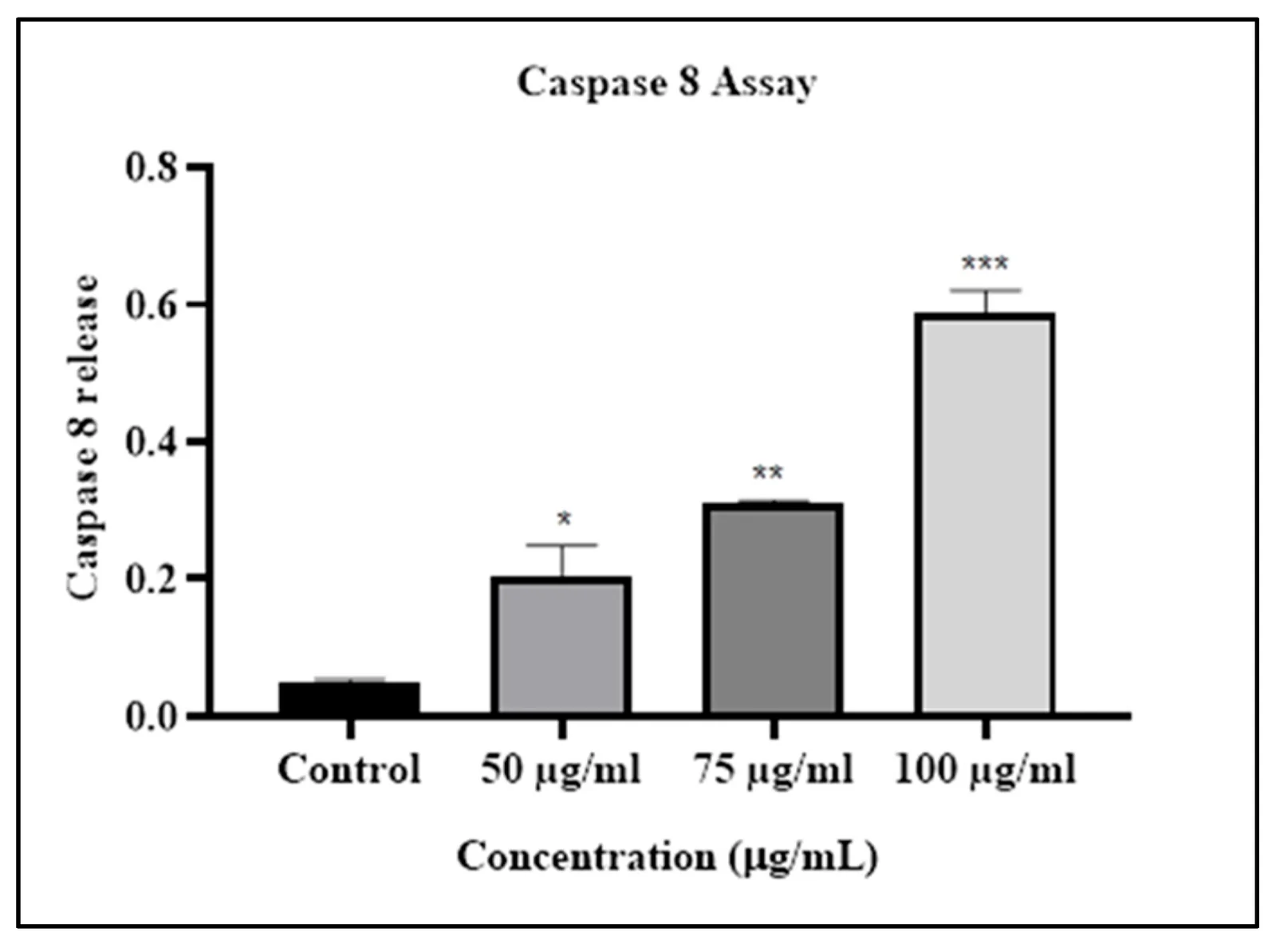
Caspase-8 activity in ZnO-NPs-treated Saos-2 cells. Treatment with biogenic ZnO-NPs significantly increased caspase-8 activity in Saos-2 osteosarcoma cells compared with the untreated control. Data are represented as mean ± SD (*n* = 3). Statistical significance was determined using one-way ANOVA followed by Dunnett’s multiple comparison test. * *p* < 0.05, ** *p* < 0.01, *** *p* < 0.001 compared with control.

Caspases serve as the major activators of apoptosis in both intrinsic and extrinsic signaling pathways. The levels of caspase-8 involved in extrinsic signaling pathways were analyzed. Figure 8 depicts that caspase-8 was only slightly activated in the control group. Conversely, the activity of caspase-8 was elevated on increasing dosages (50, 75, and 100 μg mL^−1^) of ZnO-NPs.

### 3.9. Flow Cytometric Analysis

**Figure 9:**
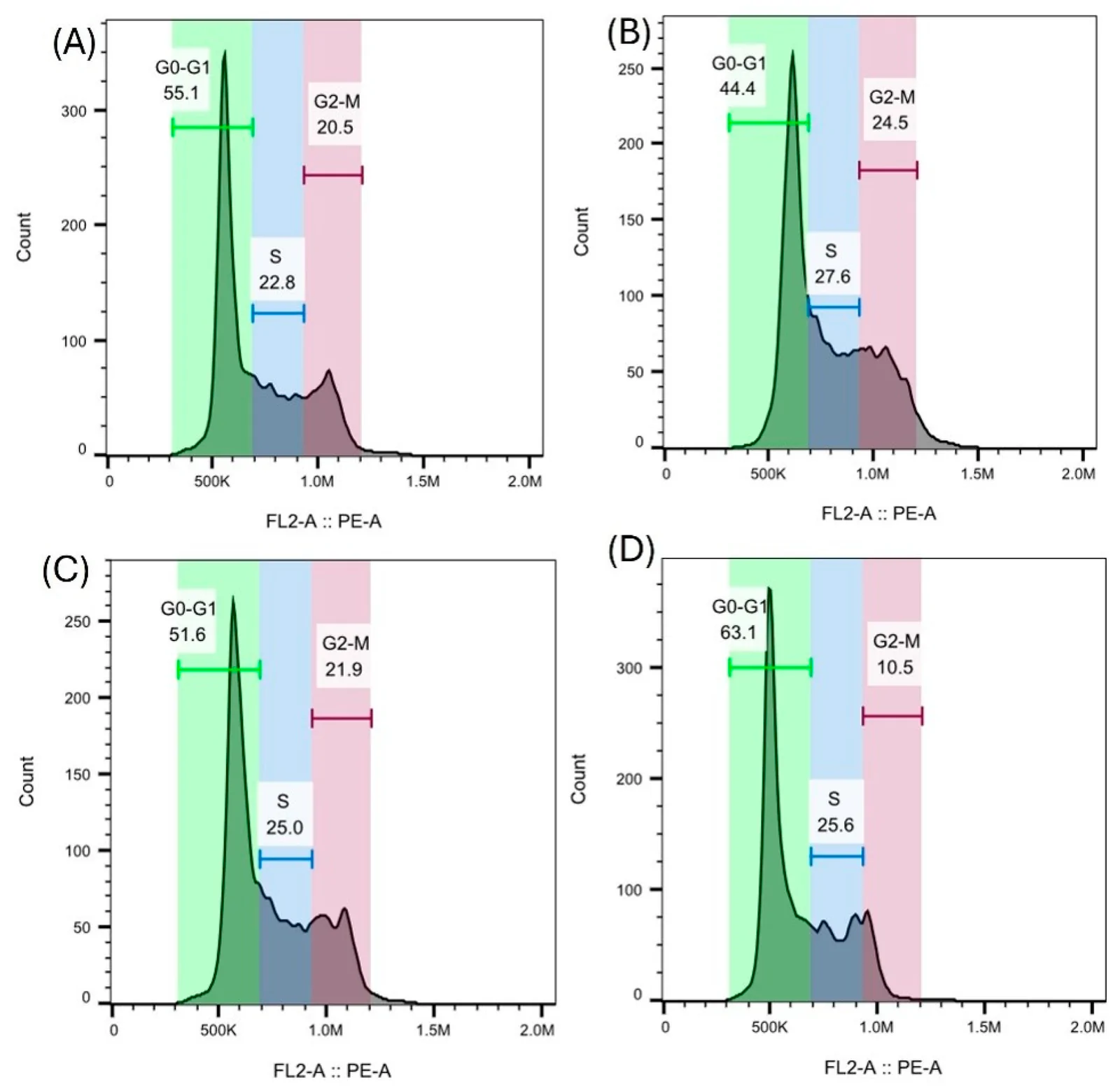
Cell-cycle distribution of Saos-2 cells after ZnO-NPs treatment. Flow cytometry analysis of (**A**) Control, (**B**) 50, (**C**) 75, and (**D**) 100 μg mL^−1^ of ZnO-NPs treatments demonstrated ZnO-NP-induced alterations in cell-cycle progression, with increased G0/G1-phase accumulation at the highest treatment concentration compared with untreated control cells.

Cell-cycle was analyzed through flow cytometry, based on DNA content analysis to evaluate the anti-proliferative effect of ZnO-NPs on Saos-2 cells. Cell-cycle distribution was analyzed by flow cytometry following a sequential gating strategy. Cellular debris was excluded using forward scatter (FSC) and side scatter (SSC) parameters, while doublets and cell aggregates were eliminated through pulse geometry analysis to ensure accurate DNA content measurements. It can be noted from Figure 9 that the control group exhibited G0/G1 population 55.1%, whereas the ZnO-NPs (50 μg mL^−1^) decreased the G0/G1 population to 44.4% in the S phase (27.6%) and G2/M phase (24.5%), demonstrating disruption of normal cell-cycle progression. At 75 μg mL^−1^, cells showed G0/G1 population (51.6%), with 25.0% and 21.9% of cells distributed in the S and G2/M phases, respectively. At highest dosage (100 μg mL^−1^) proportion of cells in the G0/G1 phase elevated to 63.1%, with notable reduction in the G2/M population to 10.5%, while the S-phase demonstrated 25.6%. The accumulation of cells in the G0/G1 phase suggests that the ZnO-NPs induce G0/G1 cell-cycle arrest and suppress the proliferation of Saos-2 cells.

### 3.10. Gene Expression Studies of Bcl-2, Bax, and p53

**Figure 10:**
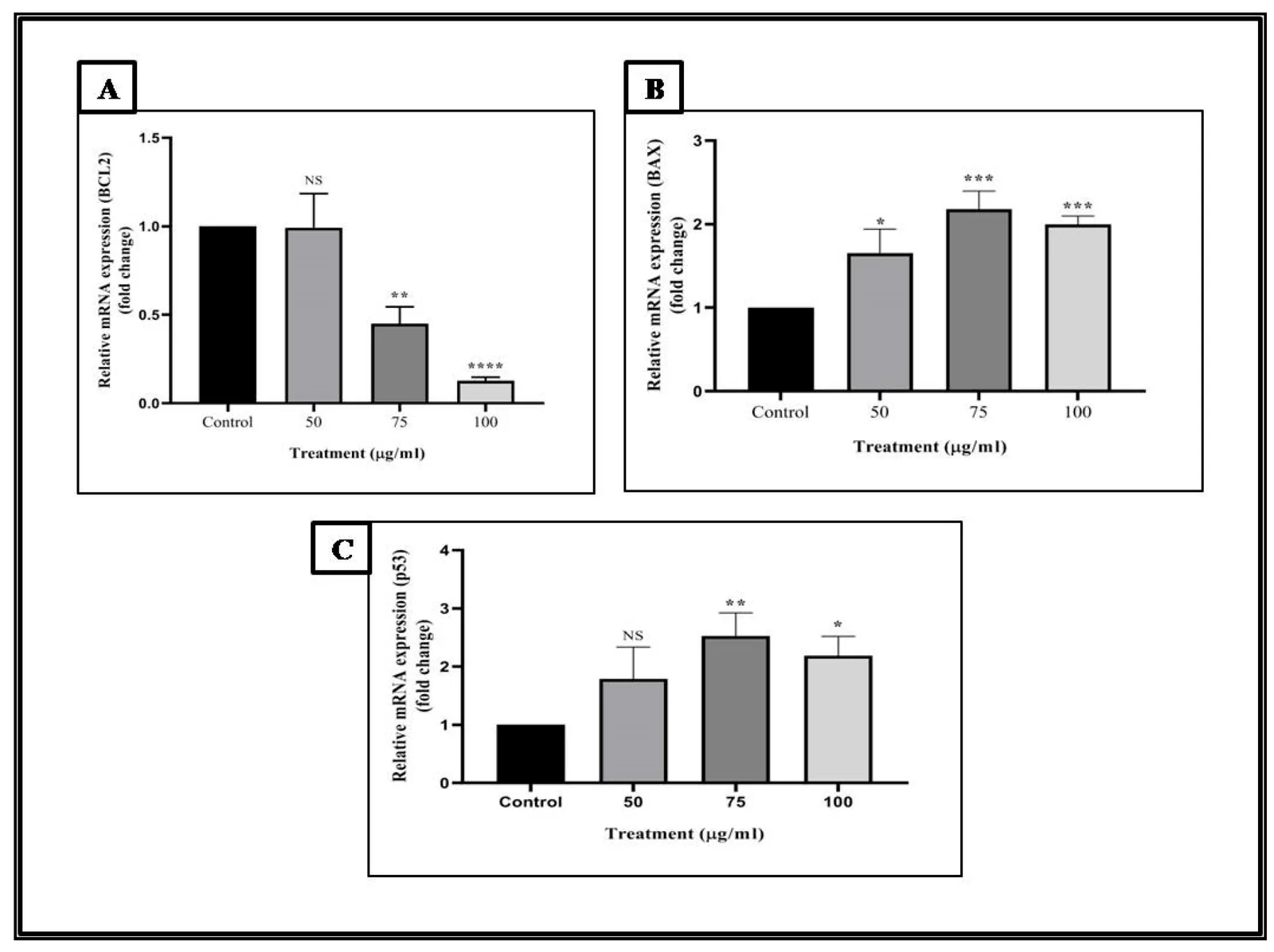
Effect of ZnO-NPs on gene expression in Saos-2 cells. (**A**) Bcl-2, (**B**) Bax, and (**C**) p53 gene expression levels after ZnO-NPs treatment. Relative mRNA expression was determined using quantitative real-time PCR. Data are denoted as mean ± SD (n = 3). Statistical significance was determined relative to the control group using one-way ANOVA followed by Dunnett’s multiple comparison test. * *p* < 0.05, ** *p* < 0.01, *** *p* < 0.001, **** *p* < 0.0001; NS, not significant compared with control.

To comprehend the molecular alterations in Saos-2 cells following ZnO-NPs treatment, the expression of apoptotic and anti-apoptotic markers was examined as depicted in Figure 10A–C. The expression of anti-apoptotic marker Bcl-2 declined in treated groups compared to controls. At doses of 50, 75, and 100 μg mL^−1^, the groups treated with ZnO-NPs exhibited elevated expression of the apoptotic markers Bax and p53. With a deeper look, the expression of both Bax and p53 was greater at a concentration of 75 μg mL^−1^.

## 4. Discussion

Cancer is a multifaceted disease that results from environmental, genetic, and infectious causes. Osteosarcoma is a primary malignant bone tumor that affects 3.4 million people globally on an annual basis. Conventional therapies for osteosarcoma are neoadjuvant and adjuvant chemotherapy, radiotherapy, and surgery, which are unsuccessful in certain cases [1]. Indeed, novel nanoparticle-based approaches have been designed to mitigate the adverse implications of conventional treatments and anticancer medications. In this context, the discipline of “phyto nano-oncology,” which integrates oncology, nanotechnology, and medicinal plants, is opening up new and promising avenues for the treatment of cancer. Medicinal plants are a great source of therapeutic molecules with the potential to treat cancer; additionally, they can also be employed to synthesize metal or metal oxide NPs in reliable and economically feasible ways [27]. Accordingly, the current study examines the anti-tumor effect of biogenically produced ZnO-NPs by employing *Ficus benghalensis* bark extract against osteosarcoma Saos-2 cells.

ZnO-NPs produced via the phytogenic method offer additional advantages over physical and chemical methods, such as being solely dependent on phytochemical constituents like flavonoids, alkaloids, phenols, and other phytocompounds. In recent years, ZnO-NPs have drawn notable attention in the field of cancer therapy. ZnO-NPs demonstrate great applications in diagnosis and are widely utilized as a desirable anticancer agent against a range of cancers such as liver, colon, lung, breast, etc. Concisely, ZnO-NPs are a promising platform for cancer treatments because of their nano size, surface area-to-volume ratio, excitation energy, wide direct band gap, and biocompatibility [27,28].

Prior to examining ZnO-NPs’ cytotoxicity, their physicochemical properties were studied. The FTIR analysis exhibited absorption peaks responsible for O-H stretching vibrations (3442 cm^−1^), denoting the existence of hydroxyl groups or adsorbed water. There are peaks at 2998, 1366, and 922 cm^−1^ owing to C-H stretching and bending vibrations, suggesting the presence of organic residues or surface-adsorbed hydrocarbons. The 2319 cm^−1^ peak is due to CO_2_ or CO stretching vibrations; the 1601 cm^−1^ peak corresponds to O-H bending or C=O stretching vibrations, implying surface carbonation or adsorbed organic molecules. The peak at 1026 cm^−1^ is due to C-O stretching vibrations, indicating possible organic compounds. The peaks at 647 and 635 cm^−1^ are characteristic of Zn-O stretching vibrations, thus confirming ZnO as the primary component [29]. The zeta potential value of ZnO-NPs was approximately +5 to +10 mV, indicating the occurrence of a major peak near neutrality, suggesting relatively low surface charge. Furthermore, zeta potential values exceeding ±30 mV are indicative of strong electrostatic stabilization, whereas values close to 0 mV are associated with lowered repulsive forces and a preference for particle aggregation. The findings of zeta potential values suggest that ZnO-NPs may demonstrate decreased electrostatic stability. Despite the low zeta potential, ZnO-NPs displayed significant biological activity in our study, emphasizing that stability may not depend completely on electrostatic repulsion. Several studies reported that in the case of green-synthesized NPs, phytochemicals adsorbed on the nanoparticle surface can provide strict stabilization, thereby reducing aggregation even when zeta potential values are low [30,31,32,33]. The rod-like structures and uneven aggregation of ZnO-NPs observed in FESEM analysis are mainly due to the existence of secondary metabolites in the bark extract of *Ficus benghalensis* [16]. The EDAX pattern revealed the peaks of zinc in the major form along with oxygen and carbon, indicating the purity of ZnO-NPs [34,35]. The outcomes of particle size distribution were well correlated with zeta potential [36]. The detected peaks in XRD are consistent with the JCPDS card number 75-0576, indicating a hexagonal wurtzite phase of ZnO [15]. The identified peaks at specific 2θ angles correspond to particular lattice planes, further confirming the crystalline nature and phase purity of the ZnO-NPs [14]. The UV–Vis absorption spectrum of ZnO-NPs was found at the peak of 366 nm. Using Planck’s constant, the corresponding band gap energy was calculated to be 3.39 eV [37]. This band gap energy has significant implications for the biological applications of ZnO-NPs.

The cytotoxic potency of ZnO-NPs on Saos-2 cells was investigated by MTT assay. ZnO-NPs IC_50_ was observed at the dosage of 75 μg mL^−1^, which demonstrated 49.36% inhibition. The findings revealed that higher doses of ZnO-NPs exhibited potent antiproliferative activity on Saos-2 cells. Furthermore, plain *Ficus benghalensis* bark extract did not exert any cytotoxic activity towards Saos-2 cells; its intrinsic anticancer property appears to be insufficient to produce cytotoxicity against osteosarcoma cells. Comparatively, Doxorubicin was used as a chemotherapeutic standard to assess the responsiveness of Saos-2 cells to anticancer treatment. The suppression of cell proliferation in a dosage-dependent manner further emphasizes the anticancer efficacy. Eventually, the biocompatibility of the synthesized ZnO-NPs was evaluated using normal human bronchial epithelial (BEAS-2B) cells. The notable reduction in viability was observed only at the highest concentrations, with 70% and 60% at 200 and 400 µg mL^−1^, suggesting cytocompatibility toward non-cancerous epithelial cells. This selective safety is critically important for anticancer nanomaterials. The preferential toxicity toward malignant cells while reducing adverse effects on normal tissues makes ZnO-NPs a promising anticancer drug [6,38]. A trypan blue viability assay was performed to further validate the MTT results. The viable cell counts in the treated groups declined with increasing doses, indicating a dosage-dependent anticancer effect of ZnO-NPs. The phase contrast microscopic images of ZnO-NPs-treated Saos-2 cells displayed rounding and shrinkage of cells and detached cells with disoriented structures. In comparison with the morphology of the control, the treated cells had disrupted cell membranes, confirming the cytotoxicity of ZnO-NPs. In the DAPI staining, the intensity of blue fluorescence in cells increased with increasing dosages, indicating that the condensed nuclei are where the DAPI dye specifically binds. The images of treated Saos-2 cells in morphological analysis and DAPI staining revealed the existence of shrunken cells, nuclear condensation, and detachment of cells from the surface, which are the hallmarks of apoptosis [39]. Apoptosis is regarded as a major anticancer mechanism, which includes the activation of a number of molecular events that result in cell death with cellular, morphological, and biochemical alterations [40]. Therefore, it can be established from the experiments above that ZnO-NPs induce morphological alterations that promote apoptosis.

To explore more about the anticancer strategy of ZnO-NPs, oxidative stress markers and antioxidants were analyzed. Oxidative stress is the primary strategy for the cause of cancer cell death mediated by NPs. Reactive oxygen species disrupt the intracellular equilibrium between oxidative stress and antioxidant systems, resulting in cellular damage [41]. The biogenic ZnO-NPs significantly increased intracellular ROS generation, nitric oxide production, and lipid peroxidation in Saos-2 cells, while pre-treatment with NAC markedly attenuated these effects. These results strongly suggest that oxidative stress is crucial in ZnO-NPs-induced cytotoxicity. The excessive ROS accumulation observed following ZnO-NPs exposure may result from nanoparticle internalization, causing disruption of cellular redox homeostasis. Additionally, increased ROS stimulates nitric oxide production and the formation of reactive nitrogen species, thereby intensifying oxidative damage. Consistent with this mechanism, NAC-mediated ROS scavenging significantly reduced NO levels, indicating an interconnection between oxidative and nitrosative stress pathways. Similar ROS-dependent anticancer effects of ZnO nanoparticles have been reported in several cancer models, where oxidative stress was identified as a critical trigger of apoptosis and growth inhibition. The increase in lipid peroxidation confirms oxidative damage to cellular membranes. ROS-mediated oxidation of membrane lipids compromises membrane integrity, modifies mitochondrial function, and leads to apoptotic signaling. The significant reduction in lipid peroxidation following NAC pre-treatment demonstrates that membrane damage is ROS-dependent. These findings are in agreement with previous studies demonstrating that ZnO-NPs induce oxidative stress-mediated apoptosis through mitochondrial dysfunction and redox imbalance [42,43,44]. On the other hand, the expressions of antioxidants such as catalase, which regulates hydrogen peroxide to water, SOD, which converts highly reactive O2- to harmless hydrogen peroxide, and GSH, a significant antioxidant tripeptide, declined in ZnO-NPs-treated cells due to oxidative stress and overload of reactive oxygen species. The increased levels of oxidative stress markers also caused inactivation of antioxidants; hence, their expressions were reduced [10,45]. Generally, the development of cancer is linked to the disruption of checkpoint controls, which govern the normal cell cycle, including DNA integrity and gene expression. The outcomes of flow cytometry DNA content analysis exhibited that ZnO-NPs induced accumulation of Saos-2 cells in the G0/G1 phase, increasing the G0/G1 population, which is consistent with the reduced cell viability observed in the MTT assay. Consequently, it indicates that elevated dosages of ZnO-NPs increasingly inhibit cell-cycle advancement, ultimately preventing the effective transition from G1 to later phases. Additionally, ZnO-NPs are known to induce ROS-mediated DNA damage, activating the ATM/ATR–p53–p21 signaling pathway, which eventually results in the arrest of cell-cycle progression and causes apoptosis under sustained oxidative stress. Similar G0/G1 arrest has been reported in osteosarcoma and other cancer cells following ZnO-NP exposure, accompanied by downregulation of cyclin D1/CDK4/CDK6 and upregulation of p21 and p27, thereby causing their antiproliferative activity [46,47]. Collectively, these findings suggest that the anticancer activity of ZnO-NPs is mediated through ROS-induced cell-cycle arrest preceding apoptosis [48].

Lastly, to understand the underlying strategy of ZnO-NPs concerning its anticancerous potential at the molecular level, the expression of caspase-8, apoptotic and anti-apoptotic markers was studied. Caspases are apoptosis-linked proteins, whose activation initiates a chain of signaling events that causes apoptosis [49]. In our study, the upregulation of caspase-8 (an initiator caspase) was observed in ZnO-NPs-treated cells in a dosage-response manner, indicating the cleavage of caspase by ZnO-NPs. Bax and Bcl-2 are pro- and anti-apoptotic proteins that have contrasting impacts on the mitochondria [50]. The current study revealed that Bax and p53 are up-regulated, while Bcl-2 was down-regulated. A disparity in the Bax/Bcl-2 ratio prompts the mitochondria to be permeabilized, releasing apoptotic proteins such as cyt C, eventually resulting in cellular death. It has been proposed that the key component of the intrinsic path for stimulating mitochondrial dysfunction is the elevated expression level of Bax. Furthermore, ZnO-NPs caused considerable cellular damage that stimulated up-regulation of p53, which in turn causes overexpression of Bax and inhibits the function of the anti-apoptotic gene Bcl-2 [50,51,52]. It can be stated from the findings that ZnO-NPs induce apoptosis through a mitochondrial-mediated signaling pathway. Our results suggest that ZnO-NPs trigger cancer cell death in several ways. Since we synthesized the ZnO-NPs using a phytogenic method using bark extract of *Ficus benghalensis*, which also possesses phytocompounds with notable anticancer properties, further enhancing the ability of ZnO-NPs to inhibit the proliferation of Saos-2 cells [17]. Overall, ZnO-NPs fabricated biogenically exert a remarkable anticancer effect on Saos-2 cells.

## 5. Conclusions

In conclusion, biogenic zinc oxide nanoparticles (ZnO-NPs) fabricated using *Ficus benghalensis* bark extract demonstrated significant physicochemical characteristics consistent with nanoscale ZnO, with a hexagonal wurtzite crystalline structure, rod-like morphology, a characteristic UV–Vis absorption peak, and surface functional groups associated with phytochemical capping, which possess factors that are critical for biological interactions. The synthesized ZnO-NPs exhibited dosage-dependent anti-proliferative effects against Saos-2 osteosarcoma cells mediated by reduced cell viability, morphometric deformities, nuclear condensation, and cell shrinkage, associated with elevated levels of oxidative stress markers, depletion of antioxidant defense, activation of caspase-8, and G0/G1-phase cell-cycle arrest. Gene expression analysis further indicated increased expression of Bax and p53 and decreased expression of Bcl-2, suggesting the involvement of apoptosis-linked pathways. Collectively, the outcomes indicate that the anticancer activity of the biogenic ZnO-NPs may be mediated through oxidative stress generation, disruption of redox homeostasis, cell-cycle arrest, and activation of apoptotic signaling pathways. Nonetheless, the molecular interactions underlying these effects should be further investigated. The study did not assess long-term toxicity, biodistribution, or in vivo efficacy. Hence, future studies should include an in-depth analysis of physicochemical standardization and nanoparticle validation in animal models of osteosarcoma. Such investigations will be crucial for assessing the translational relevance and safety of biogenic ZnO-NPs.

## Data Availability

Data will be available upon reasonable request.

## Abbreviations

The following abbreviations are used in this manuscript: ZnO NPs—Zinc oxide nanoparticles, FTIR—Fourier transform infrared spectroscopy, SEM—Scanning electron microscopy, XRD—X-ray diffraction, PSA—Particle size analysis, qRT-PCR—Quantitative real-time PCR, GRAS—Generally Recognized as Safe, NSCLC—Human non-small cell lung cancer, NO—Nitric oxide, LPO—Lipid peroxidation, ROS—Reactive oxygen species, GSH—Reduced glutathione, NAC—N-acetylcysteine, DAPI—4′,6-Diamidino-2-phenylindole, DMSO—Dimethyl Sulfoxide.
